# Screening for methamphetamine and amphetamine use in trauma: a public health opportunity

**DOI:** 10.1093/haschl/qxag014

**Published:** 2026-01-20

**Authors:** Jordan M Rook, Catherine Juillard, Danielle Newton, Allison D Rosen, Serge Ngekeng, Vickie M Mays, Steven J Shoptaw

**Affiliations:** Department of Surgery, David Geffen School of Medicine at UCLA, Los Angeles, CA 90095, United States; Department of Health Policy and Management, UCLA Fielding School of Public Health, Los Angeles, CA 90095, United States; Department of Surgery, David Geffen School of Medicine at UCLA, Los Angeles, CA 90095, United States; Department of Surgery, David Geffen School of Medicine at UCLA, Los Angeles, CA 90095, United States; Department of Family Medicine, David Geffen School of Medicine at UCLA, Los Angeles, CA 90095, United States; Sustainable Program for Trauma Research, Education, and Mentorship (STREaM), University of Buea, Cameroon; Department of Health Policy and Management, UCLA Fielding School of Public Health, Los Angeles, CA 90095, United States; Department of Psychology, UCLA, Los Angeles, CA 90095, United States; Department of Family Medicine, David Geffen School of Medicine at UCLA, Los Angeles, CA 90095, United States; Department of Psychiatry and Biobehavioral Sciences, David Geffen School of Medicine at UCLA, Los Angeles, CA 90095, United States

**Keywords:** trauma, methamphetamine, amphetamines, substance use, substance use screening, drug screening, trauma, trauma centers, injury

## Abstract

**Introduction:**

Despite the association of meth/amphetamine use with risk for unintentional injury and interpersonal violence, screening for meth/amphetamine use among injured patients is not required at US trauma centers.

**Methods:**

Using data from the American College of Surgeons Trauma Quality Programs dataset, we examined the prevalence of meth/amphetamine positivity on urine drug screen and its association with clinical outcomes at 873 trauma centers.

**Results:**

Of 4.5 million adult trauma patients, 1.5 million (33%) received a urine drug screen, of which 11% were positive for meth/amphetamines. Rates of positivity increased from 9% in 2017 to 12% in 2022. Prevalence among those screened varied widely across trauma centers (interquartile range 4%-16%). Patients who screened positive for meth/amphetamine presented with more severe injuries and required longer hospitalizations.

**Conclusion:**

Our findings indicate that meth/amphetamine use is common among trauma patients, with a prevalence several times higher than national averages. For this population, improved screening and intervention for meth/amphetamine use disorder at US trauma centers may represent an opportunity to prevent reinjury and improve the health of patients and their communities.

## Teaser

Little is known regarding the prevalence and implications of meth/amphetamine use among patients presenting for care at US trauma centers. In this retrospective cohort study of nearly 1.5 million trauma patients who received a urine drug screen, meth/amphetamines were detected in over 11%, with a pattern of endemic meth/amphetamine use across the United States. Meth/amphetamine use was associated with increased injury severity and longer hospitalizations. These findings indicate that meth/amphetamine use is common among injured patients. Improved screening and intervention for meth/amphetamine use disorder at US trauma centers is needed. These programs could address a significant risk factor for injury and improve the health of patients and their communities.

- Among 1.5 million trauma patients who were urine drug screened, 11% tested positive for methamphetamine or amphetamines.
- Prevalence of meth/amphetamine use has risen in recent years, with evidence of endemic use across trauma centers.
- Meth/amphetamine use was associated with more severe injuries and longer hospitalizations.

## Introduction

Trauma is the leading cause of death for individuals 45 years or younger in the United States. Substance use contributes to many of these injuries, with up to one-half of hospitalized trauma patients reporting a comorbid substance use disorder.^1-3^ Use, misuse, and addiction to psychoactive substances, through their direct effects on brain and behavior and their links to negative social and environmental factors, enhance risk for unintentional injury and interpersonal violence.^4-6^ In particular, meth/amphetamine is strongly linked to risk for unintentional injury and injury due to interpersonal violence.^4,7-9^ Methamphetamine and amphetamine (methamphetamine metabolizes to d-amphetamine in the first pass) are both potent central nervous system stimulants that produce euphoria, disinhibition, agitation, and in a minority of individuals, meth/amphetamine-induced psychosis. Prior studies have noted the prevalence of methamphetamine use to be higher than other stimulant use among injured patients and several times higher relative to the general population.^8,10,11^ Despite this, among injured patients treated at American College of Surgeons (ACS) verified trauma centers, there is no screening requirement for any substance other than alcohol.^2,12^

The absence of protocolized screening for meth/amphetamine may be a missed medical and public health intervention opportunity. This is particularly salient given that national rates of methamphetamine use have increased in recent years, particularly in the West and Midwest.^10^ To our knowledge, there are few research efforts exploring how substance use screening in trauma can improve downstream outcomes for injured patients with meth/amphetamine use disorders.

Given these national trends, we hypothesized that the prevalence of meth/amphetamine use among injured patients has risen in recent years. Furthermore, given the regionalization of meth/amphetamine availability, we hypothesized that the prevalence of meth/amphetamine use would be clustered at certain trauma centers (eg, those in areas where meth/amphetamine is endemic). To evaluate these hypotheses, we conducted an analysis of a national cohort of trauma patients to determine (1) the prevalence of meth/amphetamine use as detected by urine drug screen; (2) variation in meth/amphetamine use across trauma centers; and (3) the association of meth/amphetamine use with clinical outcomes.

## Methods

### Data source and sample

This retrospective cohort study used the 2017 to 2022 ACS Trauma Quality Programs (TQP) dataset.^13^ Each year, this national registry includes over 1 million encounters for injured patients receiving care at over 800 participating trauma centers, including most ACS-verified trauma centers and many non-ACS-verified centers. All variables are collected based on National Trauma Data Standard specifications by trained trauma registrars at each participating trauma center with data quality monitored by the ACS Committee on Trauma.^14,15^ We included all patients aged 18 and older. Patients were excluded if missing any covariate (*n* = 859 142) or outcome (*n* = 108 920) comprising 17.4% of the sample (Figure S1). This study was deemed exempt by the Institutional Review Board (IRB#23-000359) and followed the Strengthening the Reporting of Observational Studies in Epidemiology guidelines.

### Exposure

We assessed the primary exposure of meth/amphetamine use defined as methamphetamine and/or amphetamine detected on urine drug screen. We assessed methamphetamine and amphetamine use as a combined binary variable given their close link in pharmacology and pharmacokinetics.^16,17^ Given that lisdexamfetamine (ie, Vyvanse; a common treatment for attention deficit/hyperactivity disorder [ADHD]) metabolizes to amphetamine, we coded patients with comorbid ADHD and only amphetamine detected in their urine as being meth/amphetamine negative (*n* = 4616; 2.8% of meth/amphetamine positive patients). Prescribed methamphetamine (ie, Desoxyn) is rarely used for the treatment of ADHD in adults, and thus patients with ADHD and any methamphetamine positivity were considered meth/amphetamine positive cases. The presence of comorbid ADHD is a specified binary variable in the TQIP dataset. Trauma registrars review all patients' medical records and document this as positive if any diagnosis of ADHD is noted.

### Outcomes

We assessed the outcomes of injury severity score (ISS), mortality, and hospital length of stay. ISS was defined as a discrete variable ranging from zero, indicating no injury, to 75, indicating the most severe injury possible. ISS has been used extensively in trauma research as a predictor of mortality across injury mechanisms.^18,19^ Overall mortality was defined as a binary variable and included any death prior to discharge. This included patients who died while receiving care in the emergency department (ED). Hospital length of stay was measured in days as a continuous variable.

### Covariates

We evaluated the patient-level covariates of age, race and ethnicity, biological sex, health insurance, injury mechanism, injury intent, Glasgow Coma Scale, and positivity for other substances (alcohol, cannabis, ecstasy, and/or phencyclidine [PCP]). Reporting of race and ethnicity data follows the National Trauma Data Standard and reflects patient self-identification, or if incapacitated, identification by next of kin.^15^ Categories included Hispanic, non-Hispanic American Indian, non-Hispanic Asian, non-Hispanic Black, non-Hispanic Pacific Islander, non-Hispanic White, and Other. Other race and ethnicity included individuals who self-identified as “other.” Trauma mechanism and intent are derived from International Classification of Diseases, 10th Revision Cause of Injury Codes and included motor vehicle collisions, motorcycle crashes, auto vs pedestrian incidents, bicycle crashes, stabbings, firearm injuries, bite wounds, machinery-related injuries, and other injury mechanisms.^15^ Trauma intent included unintentional, self-inflected, assault, and undetermined injury intent. Alcohol positivity was evaluated as a categorical variable with the options of negative, any intoxication, and not tested. This was derived from the results of serum blood alcohol concentration.

We used a unique identifier for each trauma center to conduct analyses at the hospital-level. This identifier is maintained across years, enabling analyses of hospital-specific trends over time. We evaluated the facility-level covariates of urine drug screening frequency (defined as the proportion of injured patients who are screened at each trauma center), annual trauma volume, ACS verification level, hospital size, teaching status, and minority-serving hospital status. Minority-serving hospitals were defined as trauma centers at which 50% of injured patients identified as Black or Hispanic, consistent with prior studies.^20,21^

Given that rates of meth/amphetamine use are higher among people experiencing homelessness, we generated a binary variable representing housed vs unhoused.^22^ This variable was made available in the TQP dataset starting in 2021, as such analyses using this variable were restricted to a reduced sample including only patients treated in 2021 and 2022.

### Statistical analyses

We conducted all analyses using Stata, version 17.0 (StataCorp). We utilized the Bonferroni method to adjust significance levels for multiple hypothesis testing resulting in an α-level of 0.017.^23^ We utilized chi-squared tests and Wilcoxon Rank-Sum tests for bivariate analyses.

To evaluate our hypothesis regarding meth/amphetamine use, we generated line graphs depicting annual prevalences of urine drug screening and meth/amphetamine use from 2017 to 2022. We then used logistic regression to evaluate trends over time. β coefficients were converted to average marginal effects. The prevalence of meth/amphetamine use was calculated only among individuals who underwent biochemical drug screening.

To address our hypothesis regarding the regionalization of meth/amphetamine use, we began by excluding trauma centers in the bottom decile of overall trauma volume (<105 patients per institution). Given their low trauma volumes, estimates of meth/amphetamine use prevalence could be unreliable and subsequently bias hospital-level comparisons. We then ranked the remaining 784 trauma centers by prevalence of meth/amphetamine use and stratified trauma centers into quartiles. We used Wilcoxon-Rank Sum and chi-squared tests to compare hospitals in the first (lowest) quartile vs the fourth (highest) quartile on patient- and facility-level characteristics.

We used multivariable logistic regression to assess the association of injury characteristics with the likelihood of detecting meth/amphetamine on urine drug screen. Adjustment variables were selected based on review of prior literature and a pre-specified conceptual model.^8,24,25^ We separately assessed injury intent and injury mechanism given the potential for bias due to collinearity between these variables. Sociodemographic variables, including age, race, sex, and health insurance, were included in these models but were not reported given potential bias in interpreting these confounders. To assess the association of meth/amphetamine use and clinical outcomes, we used negative binomial regression to evaluate ISS and hierarchal linear and logistic regression models to assess length of stay and mortality, respectively. Hierarchical models use random effects to nest patients within hospitals to better evaluate the effect of individual predictors on outcomes.^24,26^ β coefficients were converted to average marginal effects. Lastly, to understand the patient and injury characteristics associated with the likelihood of receiving a urine drug screen, we used a hierarchical linear probability model to assess characteristics associated with receipt of a urine drug screen.

### Sensitivity analyses

To assess the robustness of our findings, we conducted several additional analyses. First, we assessed the association of meth/amphetamine positivity with clinical characteristics and outcomes with additional adjustment for housing status. There was no significant change in the direction nor magnitude of associations. Second, we conducted analyses comparing those who were excluded for missing data against those in the analytic sample. Furthermore, we produced estimates of meth/amphetamine use prevalence among an alternate sample, which included all participants regardless of missing data.

## Results

### Meth/amphetamine screening and prevalence

*Screening and prevalence in trauma patients.* Of 4 580 013 trauma patients across 873 trauma centers, 1 489 634 (32.5%) were urine drug screened. Screening prevalence declined from 35.0% in 2017 to 31.4% in 2022, a decrease of 0.4% points per year (95% confidence interval [95%CI] −0.4 to −0.4; *P* < .001). Of those screened, meth/amphetamine use was detected in 11.2% (*n* = 166 155), with prevalence increasing from 9.4% in 2017 to 11.9% in 2022 (β 0.6pp/year; 95%CI 0.5-0.6; *P* < .001; Table 1). Of the 166 155 patients with meth/amphetamines present on urine drug screen, methamphetamine alone was detected in 9.6% (*n* = 15 950), amphetamines alone in 73.1% (*n* = 121 422), and both methamphetamine and amphetamines in 17.3% (*n* = 28 743). Characteristics associated with receipt of screening are described in Tables S1 and S2.

**Table 1.** Characteristics of patients by presence of meth/amphetamines on urine drug screen^a^.

		Meth/Amphetamines		
Characteristic	Total	Not present	Present	*P*-value
	*N* = 1 489 634	*N* = 1 323 519	*N* = 166 115	
**Age, years,** median (IQR)	47 (31-64)	49 (31-66)	38 (30-50)	<.001
**Race and ethnicity**				
American Indian	16 303 (1.1%)	12 347 (0.9%)	3956 (2.4%)	<.001
Asian	28 646 (1.9%)	26 919 (2.0%)	1727 (1.0%)	
Black	264 282 (17.7%)	242 923 (18.4%)	21 359 (12.9%)	
Hispanic	225 189 (15.1%)	195 053 (14.7%)	30 136 (18.1%)	
Pacific Islander	40 104 (2.7%)	35 650 (2.7%)	4454 (2.7%)	
White	910 257 (61.1%)	806 927 (61.0%)	103 330 (62.2%)	
Other	4853 (0.3%)	3700 (0.3%)	1153 (0.7%)	
**Sex**				
Male	1 021 242 (68.6%)	896 106 (67.7%)	125 136 (75.3%)	<.001
Female	468 392 (31.4%)	427 413 (32.3%)	40 979 (24.7%)	
**Insurance/Payment**				
Medicaid	321 097 (21.6%)	252 019 (19.0%)	69 078 (41.6%)	<.001
Uninsured	202 919 (13.6%)	171 143 (12.9%)	31 776 (19.1%)	
Private	528 797 (35.5%)	488 506 (36.9%)	40 291 (24.3%)	
Medicare	380 222 (25.5%)	360 021 (27.2%)	20 201 (12.2%)	
Other	56 599 (3.8%)	51 830 (3.9%)	4769 (2.9%)	
**Injury mechanism**				
Fall	507 996 (34.1%)	481 544 (36.4%)	26 452 (15.9%)	<.001
Motor vehicle	560 050 (37.6%)	493 470 (37.3%)	66 580 (40.1%)	
Motorcycle	95 824 (6.4%)	82 970 (6.3%)	12 854 (7.7%)	
Auto vs Pedestrian	76 318 (5.1%)	62 670 (4.7%)	13 648 (8.2%)	
Bicycle	39 628 (2.7%)	32 288 (2.4%)	7340 (4.4%)	
Stab	82 351 (5.5%)	65 174 (4.9%)	17 177 (10.3%)	
Firearm	100 385 (6.7%)	81 733 (6.2%)	18 652 (11.2%)	
Bite	3648 (0.2%)	2719 (0.2%)	929 (0.6%)	
Other	18 531 (1.2%)	20 951 (1.5%)	2483 (1.5%)	
**Injury intent**				
Unintentional	1 245 987 (83.6%)	1 127 664 (85.2%)	118 323 (71.2%)	<.001
Self-Inflicted	36 894 (2.5%)	31 307 (2.4%)	5587 (3.4%)	
Assault	195 056 (13.1%)	155 891 (11.8%)	39 165 (23.6%)	
Undetermined	11 697 (0.8%)	8657 (0.7%)	3040 (1.8%)	
**Glasgow Coma Scale,** mean (SD)	13.7 (3.2)	13.7 (3.1)	13.5 (3.4)	<.001
**Co-intoxication**				
**Alcohol positivity**				
Negative	944 217 (63.4%)	830 261 (62.7%)	113 956 (68.6%)	<.001
Positive	383 888 (25.8%)	348 615 (26.3%)	35 273 (21.2%)	
Not tested	161 529 (10.8%)	144 643 (10.9%)	16 886 (10.2%)	
**Cannabis positivity**				
Negative	1 122 144 (75.3%)	1 027 873 (77.7%)	94 271 (56.8%)	<.001
Positive	367 490 (24.7%)	295 646 (22.3%)	71 844 (43.2%)	
**Cocaine positivity**				
Negative	1 372 630 (92.1%)	1 228 394 (92.8%)	144 236 (86.8%)	<.001
Positive	117 004 (7.9%)	95 125 (7.2%)	21 879 (13.2%)	
**Ecstasy positivity**				
Negative	1 482 303 (99.5%)	1 321 988 (99.9%)	160 315 (96.5%)	<.001
Positive	7331 (0.5%)	1531 (0.1%)	5800 (3.5%)	
**PCP positivity**				
Negative	1 477 424 (99.2%)	1 315 791 (99.4%)	161 633 (97.3%)	<.001
Positive	12 210 (0.8%)	7728 (0.6%)	4482 (2.7%)	

^a^Includes 873 hospitals.

Abbreviations: IQR, interquartile range; PCP, phencyclidine; SD, standard deviation.

Among 542 839 patients screened in 2021 and 2022, we identified 9874 (1.8%) patients experiencing homelessness. Meth/amphetamine use was detected among 47.9% (*n* = 4734) of patients experiencing homelessness vs 11.4% (*n* = 60 916) for all other patients.

### Hospital characteristics by prevalence of meth/amphetamine use

Following stratification of trauma centers by meth/amphetamine use prevalence, the 196 centers in the first quartile reported a median meth/amphetamine use prevalence of 1.9% (IQR 1.0%-2.8%) compared to 6.0% (IQR 4.7%-7.7%) among 196 second quartile centers, 12.6% (IQR 10.8%-14.6%) among 196 third quartile centers, and 21.1% (IQR 18.7%-25.7%) among 196 fourth quartile centers (*P* < .001; Table 2). Across the 6-year study period, meth/amphetamine use prevalence increased among hospitals in the first quartile (β 0.2; 95%CI 0.2-0.3; *P* < .001) and the fourth quartile (β 0.2; 95%CI 0.1-0.3; *P* < .001).

**Table 2.** Characteristics of trauma centers stratified by meth/amphetamine prevalence, low-prevalence vs high-prevalence centers.

Trauma center characteristics	Low prevalence (first quartile)	High prevalence (fourth quartile)	*P*-value
**Trauma centers,** *n*	196 (25.0%)	196 (25.0%)	
**Meth/Amphetamine Prevalence,** median (IQR)	1.9% (1.0%-2.8%)	21.1% (18.7%-25.7%)	<.001
**Screening rate**, median (IQR)	29.1% (12.7%-48.2%)	23.6% (13.2%-41.4%)	.18
**Annual trauma volume**, median patients (IQR)	704 (325-1239)	894 (482-1483)	.008
**ACS verification level,** *n* (%)			
1	44 (22.5%)	48 (24.5%)	.96
2	50 (22.5%)	54 (27.6%)	
3	29 (14.8%)	28 (14.3%)	
Not verified	73 (37.2%)	66 (33.7%)	
**Hospital beds,** *n* (%)			
<200	39 (19.9%)	48 (24.5%)	.09
201-400	79 (40.3%)	77 (39.3%)	
401-600	37 (18.9%)	47 (24.0%)	
>600	41 (20.9%)	24 (12.2%)	
**Hospital type**			
University	62 (31.6%)	50 (25.5%)	.04
Community	91 (46.4%)	89 (45.4%)	
Nonteaching	43 (21.9%)	57 (29.1%)	
**Minority-serving hospital**			
Yes	36 (18.4%)	19 (9.7%)	.01
No	160 (81.6%)	177 (90.3%)	
**Patient characteristics**			
**Patients**, *n*	944 102 (20.6%)	1 206 044 (26.3%)	
**Age**, median (IQR)	59 (37-75)	54 (34-71)	<.001
**Sex**			
Male	560 685 (59.4%)	756 539 (62.7%)	<.001
Female	383 417 (40.6%)	449 505 (37.3%)	
**Race and ethnicity**			
American Indian	1687 (0.2%)	23 955 (2.0%)	<.001
Asian	19 950 (2.1%)	32 594 (2.7%)	
Black	185 289 (19.6%)	109 795 (9.1%)	
Hispanic	128 333 (13.6%)	192 745 (16.0%)	
Pacific Islander	30 846 (3.3%)	28 502 (2.4%)	
White	577 119 (61.1%)	811 720 (67.3%)	
Other	878 (0.1%)	6733 (0.6%)	
**Insurance**			
Medicaid	133 900 (14.2%)	240 220 (19.9%)	<.001
Uninsured	100 806 (10.7%)	115 980 (9.6%)	
Private	314 384 (33.3%)	407 153 (33.8%)	
Medicare	355 653 (37.7%)	418 654 (34.7%)	
Other	39 359 (4.2%)	24 037 (2.0%)	
**Injury mechanism**			
Fall	500 232 (53.0%)	525 605 (43.6%)	<.001
Motor vehicle	234 409 (24.8%)	364 691 (30.2%)	
Motorcycle	37 196 (3.9%)	65 143 (5.4%)	
Auto vs Pedestrian	37 257 (3.9%)	50 473 (4.2%)	
Bicycle	23 616 (2.5%)	34 974 (2.9%)	
Stab	44 102 (4.7%)	63 956 (5.3%)	
Firearm	43 181 (4.6%)	61 968 (5.1%)	
Bite	3682 (0.4%)	6008 (0.5%)	
Machinery	5477 (0.6%)	9511 (0.8%)	
Other	14 950 (1.6%)	23 715 (2.0%)	
**Injury intent**			
Unintentional	832 539 (88.2%)	1 051 269 (87.2%)	<.001
Self-Inflicted	10 122 (1.1%)	19 412 (1.6%)	
Assault	97 033 (10.3%)	128 078 (10.6%)	
Undetermined	4408 (0.5%)	7285 (0.6%)	
**ISS,** median (IQR)	8.0 (4.0-10.0)	9.0 (4.0-13.0)	<.001

Abbreviations: ACS, American College of Surgeons; ISS, Injury Severity Score; SD, standard deviation.

Compared to trauma centers with the lowest prevalence of meth/amphetamine use (first quartile), high-prevalence trauma centers (fourth quartile) had greater median annual trauma volumes (894 patients [IQR 482-1483] vs 704 patients [IQR 325-1239]; *P* = .008) and were less likely to be university-affiliated teaching hospitals (25.5% vs 31.6%; *P* = .04) or minority-serving trauma centers (9.7% vs 18.4%; *P* = .01). High-prevalence trauma centers treated a greater proportion of American Indian patients (2.0% vs 0.2%), Hispanic patients (16.0% vs 13.6%), and White patients (67.3% vs 61.1%) and less often treated Black patients (9.1% vs 19.6%; *P* < 0.001) when compared with low-prevalence centers. Patients presenting to high-prevalence centers were more often Medicaid-insured (19.9% vs 14.2%; *P* < .001) and were more likely to be injured by motor vehicle collision (30.2% vs 24.8%), firearm (5.1% vs 4.6%), and stabbing (5.3% vs 4.7%; *P* < .001).

### Characteristics and outcomes associated with meth/amphetamine use

*Other substance use.* Compared to those who screened negative, patients who screened positive for meth/amphetamines more often screened positive for cannabis (43.2% vs 22.3%; *P* < .001), cocaine (13.2% vs 7.2%; *P* < .001), ecstasy (3.5% vs 0.1%; *P* < .001), and PCP (2.7% vs 0.6%; *P* < .001). They were less likely to be co-intoxicated with alcohol (21.2% vs 26.3%; *P* < .001; Table 1).

*Injury and clinical characteristics.* Compared to those with unintentional injuries, patients with self-inflicted injuries (AOR 1.1; 95%CI 1.1-1.1), undetermined injury intent (AOR 2.4; 95%CI 2.3-2.5; *P* < .001), and injury by assault (AOR 1.8; 95%CI 1.7-1.8) were more likely to have meth/amphetamines detected. Compared to those injured in motor vehicle collisions, patients injured by auto vs pedestrian (AOR 1.6; 95%CI 1.5-1.6), bicycle crash (AOR 1.6; 95%CI 1.6-1.6), stabbing (AOR 1.5; 95%CI 1.5-1.6), firearm (AOR 1.7; 95%CI 1.7-1.7), and bite (AOR 2.1; 95%CI 1.9-2.3) were most likely to screen positive for meth/amphetamine (Table 3).

**Table 3.** Association of clinical characteristics with presence of meth/amphetamine on urine drug screen.

Characteristic (Reference)	Adjusted odds ratio (95%CI)^a^	*P*-value
**Injury Mechanism** (MVC)		
Fall	0.6 (0.6-0.6)	<.001
Motorcycle	1.0 (1.0-1.0)	.048
Auto vs Pedestrian	1.6 (1.5-1.6)	<.001
Bicycle	1.6 (1.6-1.6)	<.001
Stab	1.5 (1.5-1.6)	<.001
Firearm	1.7 (1.7-1.7)	<.001
Bite	2.1 (1.9-2.3)	<.001
Other	0.9 (0.8-0.9)	<.001
**Injury intent** (unintentional)		
Self-inflicted	1.1 (1.1-1.1)	<.001
Assault	1.8 (1.7-1.8)	<.001
Undetermined	2.4 (2.3-2.5)	<.001

^a^Results of multivariable logistic regression models separately assessing injury intent and injury mechanism with additional adjustment for age, race and ethnicity, sex, and health insurance.

Abbreviations: CI, confidence interval; MVC, motor vehicle collision.

*ISS, mortality, and hospitalization duration*. Meth/amphetamine use was associated with a 0.6-point increase in ISS (95%CI 0.6-0.7; *P* < .001) and a 0.6-day increase in hospitalization duration (95%CI 0.5-0.6; *P* < .001). Meth/amphetamine use was not associated with mortality (Table 4).

**Table 4.** Association of meth/amphetamine use with injury severity, hospital length of stay, and mortality.

Outcome	Adjusted marginal effect of meth/Amphetamine use, (95% CI)^a^	*P*-value
Injury severity score	0.6 points (0.6-0.7)	<.001
Hospital length of stay	+0.6 days (0.5-0.6)	<.001
Mortality	−0.1% points (−0.2 to 0.1)	.32

^a^Injury severity score was assessed with multivariable negative binomial regression. Mortality and length of stay were assessed using hierarchical multivariable logistic regression for the outcomes of mortality and linear regression for length of stay. All models adjusted for race and ethnicity, sex, insurance, injury mechanism, injury intent, year, and alcohol, cannabis, cocaine, ecstasy, and phencyclidine positivity. Hierarchical models included trauma center random effects. β coefficients were converted to average marginal effects.

### Sensitivity analysis

In analyses conducted among a reduced sample of patients treated in 2021 and 2022, additional adjustment for housing status did not significantly alter the associations between injury characteristics and meth/amphetamine positivity (Table S3). For the outcomes of ISS and hospital length of stay, there was no change in the direction nor significance of associations. However, for mortality, meth/amphetamine positivity was associated with a 0.4 pp decrease in mortality (95%CI −0.6 to −0.3; Table S4). On bivariate analyses comparing the analytic cohort to those with missing data, there were significant differences across all characteristics (Table S5). Among those with missing data (*n* = 250 824; 14.4% of initial sample), meth/amphetamine use prevalence was 14.9% (*n* = 37 474) compared to 11.2% (*n* = 166 115) for the analytic sample. This produced an overall prevalence of 11.7% (*n* = 203 589) for the total sample (*n* = 1 740 458) when including those with missing data.

## Discussion

In this national study, methamphetamine and/or amphetamines were detected in one-in-nine urine drug screens performed among injured patients at US trauma centers. Despite an increase in the prevalence of meth/amphetamine use from 9% in 2017 to 12% in 2022, urine drug screening rates declined during this period. Our findings provide no indication for this discordance. Among this reduced sample of cases, meth/amphetamine use was linked to significant consequences for patients including more frequent injuries due to interpersonal violence, greater injury severity, and longer hospitalizations. These clinical implications highlight the disconnect between screening and trends in meth/amphetamine use and indicate a missed opportunity for trauma system improvement.

Findings also confirmed our hypothesis that there are meaningful differences in meth/amphetamine prevalence across hospitals. Meth/amphetamine use prevalence ranged from less than 2% across 196 trauma centers in the lowest meth/amphetamine prevalence quartile vs over 20% for the 196 trauma centers in the highest quartile. While there are no geographic identifiers in the TQP dataset, these findings indicate an endemic distribution of meth/amphetamine use among trauma patients. This is consistent with prior reports of meth/amphetamine use in the general population which have identified higher prevalences in the western United States compared to the East.^27^ The addition of a geographic identifier to the TQP dataset would aid in identifying trauma centers serving communities with endemic meth/amphetamine use such that additional support and resources can be provided to address this critical public health issue.

In this sample, over 11% of screened patients tested positive for meth/amphetamines, a higher prevalence than prior studies. Grigorian et al. reported a 3% prevalence of methamphetamine intoxication among trauma patients in their national study.^8^ However, they did not study amphetamines, which comprise a large proportion of illicit stimulants and a majority of meth/amphetamines identified in our study. Furthermore, our study included recent data, including the pandemic years of 2020 to 2022, during which rates of meth/amphetamine use increased among the general public.^6,28^ We hypothesize several causes for the high prevalence of meth/amphetamine use among injured adults. First, we noted a higher proportion of injuries due to penetrating mechanisms and assault among meth/amphetamine users. This is consistent with prior studies and may reflect the underlying prevalence of meth/amphetamine use among individuals with significant social vulnerabilities including people experiencing homelessness and those previously incarcerated.^29-32^ The amalgamation of negative social drivers that affect these individuals likely contributes to their increased risk for injury due to interpersonal violence.^8,9,29^ Second, we linked meth/amphetamine use with some unintentional injury mechanisms, including motorcycle collisions and auto vs pedestrian events, perhaps highlighting key ways cognition, decision making, and disinhibition related to use of the drug may contribute to unintentional injury.^33-36^

### Policy implications

We believe that these findings inform several necessary improvements for hospital-based trauma care. First, we recommend that the ACS require drug use screening for all injured patients during trauma center evaluation and treatment. In this study, less than one-third of injured patients received a urine drug screen. The ACS has previously encouraged the deployment of successful and impactful universal screening for alcohol use disorders by requiring alcohol screening and intervention for trauma center verification.^37^ This implementation process can serve as a valuable blueprint as these services are extended to substances other than alcohol. Furthermore, the universal deployment of screening may address patterns of biased screening identified in this cohort and in prior research.^38^ Additionally, we recommend that the ACS requirement for brief intervention for patients who screen positive for alcohol use disorder be extended to all substance use disorders, including meth/amphetamine use disorder.^39^ These encounters may present an invaluable opportunity to offer critical treatment services, as individuals with substance use disorders are more likely to engage in treatment following an injury.^40^ Thus, these hospitalizations may allow providers to connect patients with the resources necessary to help them meet their personal goals regarding meth/amphetamine use. Furthermore, with meth/amphetamine use linked to increased injury severity, risk for injury, and length of hospitalization, improved screening and intervention programs may reduce health system costs by preventing future injury-related admissions. This is particularly salient given that 60% of individuals who screened positive for meth/amphetamines were uninsured or publicly insured, indicating that health systems likely absorb some of the costs of care for this vulnerable patient population.

### Limitations

This study has several limitations. First, as described above, we assessed rates of meth/amphetamine positivity among biochemically screened trauma patients and not trauma patients overall. It is plausible that selection bias (ie, providers were more likely to screen patients who appeared under the influence of meth/amphetamines) inflated the prevalence among screened patients relative to trauma patients overall. Thus, estimates of meth/amphetamine use prevalence should not be extrapolated to all injured patients treated at trauma centers. Second, TQP does not provide any geographic identifiers. Thus, while we were able to identify significant variation across facilities, we were not able to identify the regions of the country where meth/amphetamine use was clustered. Third, the presence of comorbid ADHD is collected for the TQP dataset from patients' medical records. It is plausible that for some patients, particularly those who are severely injured and for whom there is no medical history available, this variable is falsely negative. This would produce erroneously high estimates of meth/amphetamine use incidence. Fourth, this study did not report the results of interview-based screening, an acceptable screening method per the ACS. Thus, rates of screening are likely underestimated. Fifth, we utilized complete case analysis which resulted in the exclusion of nearly 15% of the sample, potentially affecting external validity. We sought to address this by calculating the prevalence of meth/amphetamine use among an alternate sample including those with missing data. Interestingly, these cases with missing data had a higher prevalence of meth/amphetamine use. We hypothesize that this may be due to behavioral and cognitive changes associated with meth/amphetamine use which may limit data collection for some variables based on patient's self-reported history.

## Conclusions

In this national study of trauma patients, meth/amphetamines were detected in greater than one-in-nine patients who received a urine drug screen. Furthermore, we found that the prevalence of meth/amphetamine use is rising with an endemic distribution across trauma centers. With the understanding that meth/amphetamine use precipitates injuries and has severe negative consequences for patients, the implementation of high-quality universal screening and intervention may serve a vital public health need.

## Supplementary Material

qxag014_Supplementary_Data
