# Supplementary material for: Screening for methamphetamine and amphetamine use in trauma: a public health opportunity
Source: Health Aff Sch. 2026 Jan 20;4(2):qxag014. doi: 10.1093/haschl/qxag014 (PMC12898918; doi:10.1093/haschl/qxag014)
Supplement: qxag014_Supplementary_Data [file qxag014_supplementary_data.zip › Online Supplement_Revised .docx]

Supplemental Figure 1: Flowchart Describing Cohort Construction


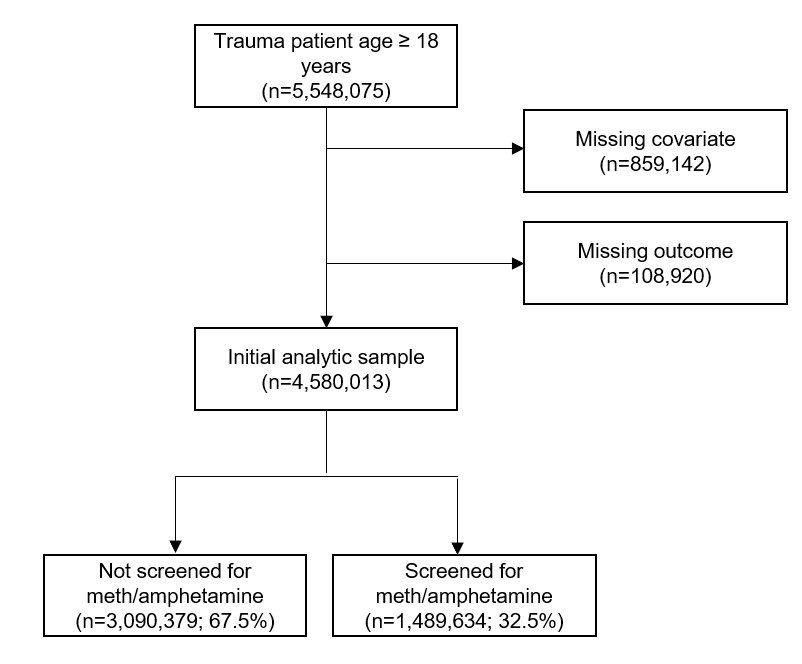


Supplemental Table 1: Characteristics of Patients by Receipt of a Urine Drug Screen

| **Characteristic** |  | **Total** |  | **Not Screened** | **Screened** | **p-value** |  |
| --- | --- | --- | --- | --- | --- | --- | --- |
|  |  | N=4,580,013 |  | N=3,090,379 | N=1,489,634 |  |  |
|  |  |  |  |  |  |  |  |
| **Age, years,** median (IQR) | | 57 (35-73) |  | 61 (39-76) | 47 (31-64) | <0.001 |  |
|  |  |  |  |  |  |  |  |
| **Race and Ethnicity** |  |  |  |  |  |  |  |
| American Indian |  | 39,341 ( 0.9%) |  | 23,038 ( 0.7%) | 16,303 ( 1.1%) | <0.001 |  |
| Asian |  | 85,816 ( 1.9%) |  | 57,170 ( 1.8%) | 28,646 ( 1.9%) |  |  |
| Black |  | 668,203 (14.6%) |  | 403,921 (13.1%) | 264,282 (17.7%) |  |  |
| Hispanic |  | 558,168 (12.2%) |  | 332,979 (10.8%) | 225,189 (15.1%) |  |  |
| Pacific Islander |  | 106,106 ( 2.3%) |  | 66,002 ( 2.1%) | 40,104 ( 2.7%) |  |  |
| White |  | 3,112,032 (67.9%) |  | 2,201,775 (71.2%) | 910,257 (61.1%) |  |  |
| Other |  | 10,347 ( 0.2%) |  | 5,494 ( 0.2%) | 4,853 ( 0.3%) |  |  |
|  |  |  |  |  |  |  |  |
| **Sex** |  |  |  |  |  |  |  |
| Male |  | 2,766,511 (60.4%) |  | 1,745,269 (56.5%) | 1,021,242 (68.6%) | <0.001 |  |
| Female |  | 1,813,502 (39.6%) |  | 1,345,110 (43.5%) | 468,392 (31.4%) |  |  |
|  |  |  |  |  |  |  |  |
| **Insurance** |  |  |  |  |  |  |  |
| Medicaid |  | 684,337 (14.9%) |  | 363,240 (11.8%) | 321,097 (21.6%) | <0.001 |  |
| Uninsured |  | 536,464 (11.7%) |  | 333,545 (10.8%) | 202,919 (13.6%) |  |  |
| Private |  | 1,538,687 (33.6%) |  | 1,009,890 (32.7%) | 528,797 (35.5%) |  |  |
| Medicare |  | 1,696,657 (37.0%) |  | 1,316,435 (42.6%) | 380,222 (25.5%) |  |  |
| Other |  | 123,868 ( 2.7%) |  | 67,269 ( 2.2%) | 56,599 ( 3.8%) |  |  |
|  |  |  |  |  |  |  |  |
| **Injury Mechanism** |  |  |  |  |  |  |  |
| Fall |  | 2,190,748 (47.8%) |  | 1,682,752 (54.5%) | 507,996 (34.1%) | <0.001 |  |
| Motor Vehicle |  | 1,317,690 (28.8%) |  | 757,640 (24.5%) | 560,050 (37.6%) |  |  |
| Motorcycle |  | 213,504 ( 4.7%) |  | 117,680 ( 3.8%) | 95,824 ( 6.4%) |  |  |
| Auto vs Pedestrian |  | 159,605 ( 3.5%) |  | 83,287 ( 2.7%) | 76,318 ( 5.1%) |  |  |
| Bicycle |  | 110,910 ( 2.4%) |  | 71,282 ( 2.3%) | 39,628 ( 2.7%) |  |  |
| Stab |  | 209,556 ( 4.6%) |  | 127,205 ( 4.1%) | 82,351 ( 5.5%) |  |  |
| Firearm |  | 237,263 ( 5.2%) |  | 136,878 ( 4.4%) | 100,385 ( 6.7%) |  |  |
| Bite |  | 21,593 ( 0.5%) |  | 17,945 ( 0.6%) | 3,648 ( 0.2%) |  |  |
| Machinery |  | 34,441 ( 0.8%) |  | 29,538 ( 1.0%) | 4,903 ( 0.3%) |  |  |
| Other |  | 84,703 ( 1.8%) |  | 66,172 ( 2.1%) | 18,531 ( 1.2%) |  |  |
|  |  |  |  |  |  |  |  |
| **Injury Intent** |  |  |  |  |  |  |  |
| Unintentional |  | 4,041,271 (88.2%) |  | 2,795,284 (90.5%) | 1,245,987 (83.6%) | <0.001 |  |
| Self-Inflicted |  | 61,463 ( 1.3%) |  | 24,569 ( 0.8%) | 36,894 ( 2.5%) |  |  |
| Assault |  | 450,411 ( 9.8%) |  | 255,355 ( 8.3%) | 195,056 (13.1%) |  |  |
| Undetermined |  | 26,868 ( 0.6%) |  | 15,171 ( 0.5%) | 11,697 ( 0.8%) |  |  |
|  |  |  |  |  |  |  |  |
| **Glasgow Coma Scale,** mean (SD) | | 14.2 (2.6) |  | 14.4 (2.2) | 13.7 (3.2) | <0.001 |  |
|  | |  |  |  |  |  |  |
| **Injury Severity Score,** median (IQR) |  | 9 (4-11) |  | 9 (4-10) | 9(5-14) | <0.001 |  |
|  |  |  |  |  |  |  |  |
| **Alcohol Positivity** |  |  |  |  |  |  |  |
| Negative |  | 1,620,860 (35.4%) |  | 676,643 (21.9%) | 944,217 (63.4%) | <0.001 |  |
| Positive |  | 640,386 (14.0%) |  | 256,498 ( 8.3%) | 383,888 (25.8%) |  |  |
| Not Tested |  | 2,318,767 (50.6%) |  | 2,157,238 (69.8%) | 161,529 (10.8%) |  |  |
|  |  |  |  |  |  |  |  |
| **ED Disposition** | |  |  |  |  |  |  |
| Discharged |  | 454,214 ( 9.9%) |  | 340,199 (11.0%) | 114,015 ( 7.7%) | <0.001 |  |
| Floor |  | 2,574,649 (56.2%) |  | 1,833,875 (59.3%) | 740,774 (49.7%) |  |  |
| ICU |  | 840,208 (18.3%) |  | 428,356 (13.9%) | 411,852 (27.6%) |  |  |
| OR |  | 534,069 (11.7%) |  | 345,669 (11.2%) | 188,400 (12.6%) |  |  |
| Transfer |  | 115,351 ( 2.5%) |  | 89,985 ( 2.9%) | 25,366 ( 1.7%) |  |  |
| Deceased |  | 45,716 ( 1.0%) |  | 41,094 ( 1.3%) | 4,622 ( 0.3%) |  |  |
| AMA |  | 15,806 ( 0.3%) |  | 11,201 ( 0.4%) | 4,605 ( 0.3%) |  |  |
|  |  |  |  |  |  |  |  |
| **ACS Verification** |  |  |  |  |  |  |  |
| Level 1 |  | 1,946,033 (42.5%) |  | 1,265,494 (40.9%) | 680,539 (45.7%) | <0.001 |  |
| Level 2 |  | 1,177,377 (25.7%) |  | 777,398 (25.2%) | 399,979 (26.9%) |  |  |
| Level 3 |  | 292,727 ( 6.4%) |  | 229,762 ( 7.4%) | 62,965 ( 4.2%) |  |  |
| Not Verified |  | 1,163,876 (25.4%) |  | 817,725 (26.5%) | 346,151 (23.2%) |  |  |
|  |  |  |  |  |  |  |  |
| **Hospital Type** |  |  |  |  |  |  |  |
| University |  | 1,916,622 (41.8%) |  | 1,229,803 (39.8%) | 686,819 (46.1%) | <0.001 |  |
| Community |  | 1,818,676 (39.7%) |  | 1,245,049 (40.3%) | 573,627 (38.5%) |  |  |
| Nonteaching |  | 844,715 (18.4%) |  | 615,527 (19.9%) | 229,188 (15.4%) |  |  |
|  |  |  |  |  |  |  |  |

Abbreviations: IQR – interquartile range; SD – standard deviation; ICU – intensive care unit; OR – operating room; AMA – left against medical advice. ACS – American College of Surgeons.

Supplemental Table 2: Association of Patient and Clinical Characteristics with Receipt of a Urine Drug Screen ^a^

| **Characteristic** (Reference) |  | **β Coefficient (95% CI)** | **p-value** |
| --- | --- | --- | --- |
|  |  |  |  |
| **Age, years** (36-50) |  |  |  |
| 18-25 |  | -0.3 (-0.5 to -0.2) | <0.001 |
| 26-35 |  | 0.4 (0.3 to 0.6) | <0.001 |
| 51-65 |  | -3.4 (-3.5 to -3.3) | <0.001 |
| 66-80 |  | -10.2 (-10.3 to -10.0) | <0.001 |
| 81+ |  | -14.7 (-14.8 to -14.5) | <0.001 |
|  |  |  |  |
| **Race and Ethnicity** (Non-Hispanic White) |  |  |  |
| American Indian |  | 4.8 (4.3 to 5.2) | <0.001 |
| Asian |  | -3.5 (-3.8 to -3.3) | <0.001 |
| Black |  | 1.0 (0.9 to 1.1) | <0.001 |
| Hispanic |  | -0.9 (-1.0 to -0.8) | <0.001 |
| Pacific Islander |  | -0.6 (-0.8 to -0.3) | <0.001 |
| Other |  | -0.8 (-1.6 to 0.0) | 0.04 |
|  |  |  |  |
| **Sex** (Male) |  |  |  |
| Female |  | -3.7 (-3.8 to -3.7) | <0.001 |
|  |  |  |  |
| **Insurance** (Private) |  |  |  |
| Medicaid |  | 5.6 (5.5 to 5.7) | <0.001 |
| Uninsured |  | 2.5 (2.4 to 2.6) | <0.001 |
| Medicare |  | -0.3 (-0.4 to -0.2) | <0.001 |
| Other |  | 1.5 (1.3 to 1.8) | <0.001 |
|  |  |  |  |
| **ED Disposition** (Discharged) |  |  |  |
| Floor |  | 12.8 (12.7 to 13.0) | <0.001 |
| ICU |  | 22.7 (22.5 to 22.9) | <0.001 |
| OR |  | 9.8 (9.6 to 10.0) | <0.001 |
| Transfer |  | 5.0 (4.7 to 5.3) | <0.001 |
| Deceased |  | -36.8 (-37.2 to -36.3) | <0.001 |
| AMA |  | 11.9 (11.3 to 12.6) | <0.001 |
|  |  |  |  |
| **Injury Mechanism** (Fall) |  |  |  |
| Motor Vehicle |  | 10.6 (10.5 to 10.7) | <0.001 |
| Motorcycle |  | 10.5 (10.3 to 10.7) | <0.001 |
| Auto vs Pedestrian |  | 12.6 (12.4 to 12.8) | <0.001 |
| Bicycle |  | 4.3 (4.0 to 4.5) | <0.001 |
| Stab |  | 4.4 (4.2 to 4.6) | <0.001 |
| Firearm |  | 5.8 (5.6 to 5.1) | <0.001 |
| Bite |  | -7.0 (-7.5 to -6.4) | <0.001 |
| Machinery |  | -8.9 (-9.3 to -8.4) | <0.001 |
| Other |  | -4.1 (-4.4 to -3.9) | <0.001 |
|  |  |  |  |
| **Injury Intent** (Unintentional) |  |  |  |
| Self-Inflicted |  | 20.1 (19.7 to 20.4) | <0.001 |
| Assault |  | 1.5 (1.4 to 1.7) | <0.001 |
| Undetermined |  | 5.6 (5.1 to 6.1) | <0.001 |
|  |  |  |  |
| **Glasgow Coma Scale** (15) |  |  |  |
| 13-14 |  | 12.3 (12.1 to 12.4) | <0.001 |
| 9-12 |  | 18.2 (18.0 to 18.4) | <0.001 |
| <8 |  | 17.5 (17.3 17.7) | <0.001 |
|  |  |  |  |
| **Injury Severity Score** (Minor, 2-8) | | |  |
| No injury, 1 |  | 1.6 (1.5 to 1.7) | <0.001 |
| Moderate, 9-15 |  | 1.7 (1.6 to 1.8) | <0.001 |
| Major, 16-25 |  | 5.6 (5.4 to 5.7) | <0.001 |
| Grave, ≥ 26 |  | 5.3 (5.1 to 5.5) | <0.001 |
|  |  |  |  |
| **ACS-Verification Level** (Level 1) |  |  |  |
| Level 2 |  | 1.1 (0.7 to 1.5) | <0.001 |
| Level 3 |  | -3.0 (-3.7 to -2.3) | <0.001 |
| Not Verified |  | -0.7 (-1.1 to -0.3) | 0.001 |
|  |  |  |  |
| **Hospital Type** (University) |  |  |  |
| Community |  | 1.1 (0.6 to 1.7) | <0.001 |
| Nonteaching |  | 1.9 (1.2 to 2.6) | <0.001 |
|  |  |  |  |

^a^ Outcomes assessed using heirarichal multivariable linear regression model. Models adjust for the variables listed above using fixed effects and trauma center using random effects Abbreviations: ICU – intensive care unit; OR – operating room; AMA – left against medical advice. ACS – American College of Surgeons.

Supplemental Table 3: Association of Patient and Clinical Characteristics with Presence of Meth/Amphetamine on Urine Drug Screen, 2021 and 2022

| **Characteristic** (Reference) |  | | **Adjusted Odds Ratio (95%CI) ^a^** | **p-value** |
| --- | --- | --- | --- | --- |
|  |  | |  |  |
| **Injury Mechanism** (MVC) | | |  |  |
| Fall |  | | 0.5 (0.5-0.5) | <0.001 |
| Motorcycle |  | | 1.1 (1.1-1.2) | <0.001 |
| Auto vs Pedestrian |  | | 1.6 (1.5-1.7) | <0.001 |
| Bicycle |  | | 1.6 (1.5-1.6) | <0.001 |
| Stab |  | | 1.5 (1.5-1.6) | <0.001 |
| Firearm |  | | 1.8 (1.7-1.8) | <0.001 |
| Bite |  | | 2.1 (1.9-2.4) | <0.001 |
| Other |  | | 0.9 (0.8-1.0) | 0.001 |
|  |  | |  |  |
| **Injury Intent** (Unintentional) | | | |  |
| Self-Inflicted | | 1.0 (1.0-1.1) | | 0.130 |
| Assault |  | | 1.8 (1.8-1.8) | <0.001 |
| Undetermined |  | | 2.3 (2.2-2.5) | <0.001 |
|  |  | |  |  |

^a^ Results of multivariable logistic regression models separately assessing injury intent and injury mechanism with additional adjustment for age, race and ethnicity, sex, health insurance, and housing status. Abbreviations: CI – Confidence Interval; MVC – motor vehicle collision.

Supplemental Table 4: Association of Meth/Amphetamine Use with Injury Severity, Hospital Length of Stay, and Mortality, 2021 and 2022

| **Outcome** |  | **Adjusted Marginal Effect of Meth/Amphetamine Use, (95% CI) ^a^** | **p-value** |
| --- | --- | --- | --- |
|  |  |  |  |
| Injury Severity Score |  | 0.7 points (0.6-0.8) | <0.001 |
|  |  |  |  |
| Hospital Length of Stay |  | +0.2 days (0.1-0.3) | <0.001 |
|  |  |  |  |
| Mortality |  | -0.4 percentage points (-0.6 to -0.3) | <0.001 |
|  |  |  |  |

^a^ Injury severity score was assessed with multivariable negative binomial regression. Mortality and length of stay were assessed using hierarchical multivariable logistic regression for the outcomes of mortality and linear regression for length of stay. All models adjusted for race and ethnicity, sex, insurance, injury mechanism, injury intent, housing status, year, and alcohol, cannabis, cocaine, ecstasy, and phencyclidine positivity. Hierarchical models included trauma center random effects. β coefficients were converted to average marginal effects.

Supplemental Table 5: Characteristics of Individuals with Missing Covariate Data Versus Analytic Cohort

|  |  |  |  | **Missing Covariate Data** | |  |
| --- | --- | --- | --- | --- | --- | --- |
| **Characteristic** |  | **Total** |  | **Not Missing** | **Missing** | **p-value** |
|  |  |  |  |  |  |  |
|  |  | N=1,740,458 |  | N=1,489,634 | N=250,824 |  |
| **Age, years,** median (IQR) |  | 47.0 (31.0-64.0) |  | 47.0 (31.0-64.0) | 48.0 (31.0-65.0) | <0.001 |
| Missing |  | 0 (0.0%) |  | 0 (0.0%) | 0 (0.0%) |  |
|  |  |  |  |  |  |  |
| **Race and Ethnicity** |  |  |  |  |  |  |
| American Indian |  | 18,503 ( 1.1%) |  | 16,303 ( 1.1%) | 2,200 ( 0.9%) | <0.001 |
| Asian |  | 31,875 ( 1.8%) |  | 28,646 ( 1.9%) | 3,229 ( 1.3%) |  |
| Black |  | 292,037 (16.8%) |  | 264,282 (17.7%) | 27,755 (11.1%) |  |
| Hispanic |  | 244,220 (14.0%) |  | 225,189 (15.1%) | 19,031 ( 7.6%) |  |
| Pacific Islander |  | 44,466 ( 2.6%) |  | 40,104 ( 2.7%) | 4,362 ( 1.7%) |  |
| White |  | 1,009,521 (58.0%) |  | 910,257 (61.1%) | 99,264 (39.6%) |  |
| Other |  | 5,353 ( 0.3%) |  | 4,853 ( 0.3%) | 500 ( 0.2%) |  |
| Missing |  | 94,483 ( 5.4%) |  | 0 ( 0.0%) | 94,483 (37.7%) |  |
|  |  |  |  |  |  |  |
| **Sex** |  |  |  |  |  |  |
| Male |  | 1,188,187 (68.3%) |  | 1,021,242 (68.6%) | 166,945 (66.6%) | <0.001 |
| Female |  | 549,320 (31.6%) |  | 468,392 (31.4%) | 80,928 (32.3%) |  |
| Missing |  | 2,951 ( 0.2%) |  | 0 ( 0.0%) | 2,951 ( 1.2%) |  |
|  |  |  |  |  |  |  |
| **Insurance** |  |  |  |  |  |  |
| Medicaid |  | 369,823 (21.2%) |  | 321,097 (21.6%) | 48,726 (19.4%) | <0.001 |
| Uninsured |  | 233,768 (13.4%) |  | 202,919 (13.6%) | 30,849 (12.3%) |  |
| Private |  | 594,112 (34.1%) |  | 528,797 (35.5%) | 65,315 (26.0%) |  |
| Medicare |  | 443,564 (25.5%) |  | 380,222 (25.5%) | 63,342 (25.3%) |  |
| Other |  | 63,248 ( 3.6%) |  | 56,599 ( 3.8%) | 6,649 ( 2.7%) |  |
| Missing |  | 35,943 ( 2.1%) |  | 0 ( 0.0%) | 35,943 (14.3%) |  |
|  |  |  |  |  |  |  |
| **Injury Mechanism** |  |  |  |  |  |  |
| Fall |  | 591,958 (34.0%) |  | 507,996 (34.1%) | 83,962 (33.5%) | <0.001 |
| Motor Vehicle |  | 626,330 (36.0%) |  | 560,050 (37.6%) | 66,280 (26.4%) |  |
| Motorcycle |  | 105,606 ( 6.1%) |  | 95,824 ( 6.4%) | 9,782 ( 3.9%) |  |
| Auto vs Pedestrian |  | 83,947 ( 4.8%) |  | 76,318 ( 5.1%) | 7,629 ( 3.0%) |  |
| Bicycle |  | 43,629 ( 2.5%) |  | 39,628 ( 2.7%) | 4,001 ( 1.6%) |  |
| Stab |  | 92,043 ( 5.3%) |  | 82,351 ( 5.5%) | 9,692 ( 3.9%) |  |
| Firearm |  | 113,190 ( 6.5%) |  | 100,385 ( 6.7%) | 12,805 ( 5.1%) |  |
| Bite |  | 4,211 ( 0.2%) |  | 3,648 ( 0.2%) | 563 ( 0.2%) |  |
| Other |  | 27,555 ( 1.6%) |  | 23,434 ( 1.6%) | 4,121 ( 1.6%) |  |
| Missing |  | 51,989 ( 3.0%) |  | 0 ( 0.0%) | 51,989 (20.7%) |  |
|  |  |  |  |  |  |  |
| **Injury Intent** |  |  |  |  |  |  |
| Unintentional |  | 1,414,874 (81.3%) |  | 1,245,987 (83.6%) | 168,887 (67.3%) | <0.001 |
| Self-Inflicted |  | 41,725 ( 2.4%) |  | 36,894 ( 2.5%) | 4,831 ( 1.9%) |  |
| Assault |  | 220,936 (12.7%) |  | 195,056 (13.1%) | 25,880 (10.3%) |  |
| Undetermined |  | 62,923 ( 3.6%) |  | 11,697 ( 0.8%) | 51,226 (20.4%) |  |
| Missing |  | 0 (0.0%) |  | 0 (0.0%) | 0 (0.0%) |  |
|  |  |  |  |  |  |  |
| **Glasgow Coma Scale,** mean (SD) | | 15.0 (14.0-15.0) |  | 15.0 (14.0-15.0) | 15.0 (14.0-15.0) |  |
| Missing | | 35,005 ( 2.0%) |  | 0 ( 0.0%) | 35,005 (14.0%) |  |
|  | |  |  |  |  |  |
| **ISS**, mean (SD) | | 11.0 (9.3) |  | 11.0 (9.2) | 10.6 (9.6) | <0.001 |
| **Co-Intoxication** |  |  |  |  |  |  |
|  |  |  |  |  |  |  |
| **Alcohol Positivity** |  |  |  |  |  |  |
| Negative |  | 1,066,012 (61.2%) |  | 944,217 (63.4%) | 121,795 (48.6%) | <0.001 |
| Positive |  | 437,989 (25.2%) |  | 383,888 (25.8%) | 54,101 (21.6%) |  |
| Not Tested |  | 206,994 (11.9%) |  | 161,529 (10.8%) | 45,465 (18.1%) |  |
| Missing |  | 29,463 ( 1.7%) |  | 0 ( 0.0%) | 29,463 (11.7%) |  |

Abbreviations: IQR – interquartile range; SD – standard deviation; ISS – Injury Severity Score
